# Molecular Targets Modulated by Fangchinoline in Tumor Cells and Preclinical Models

**DOI:** 10.3390/molecules23102538

**Published:** 2018-10-05

**Authors:** Myriam Mérarchi, Gautam Sethi, Lu Fan, Srishti Mishra, Frank Arfuso, Kwang Seok Ahn

**Affiliations:** 1 Faculty of Pharmacy, University of Paris Descartes, 75006 Paris, France; myriammerarchi@hotmail.fr; 2 Department of Pharmacology, Yong Loo Lin School of Medicine, National University of Singapore, Singapore 117600, Singapore; phcfanl@nus.edu.sg (L.F.); srishti.mishra@u.nus.edu (S.M.); 3 Stem Cell and Cancer Biology Laboratory, School of Pharmacy and Biomedical Sciences, Curtin Health Innovation Research Institute, Curtin University, Perth WA, 6009 Australia; frank.arfuso@curtin.edu.au; 4 College of Korean Medicine, Kyung Hee University, 24 Kyungheedae-ro, Dongdaemun-gu, Seoul 02447, Korea

**Keywords:** fangchinoline, cancer, apoptosis, proliferation, molecular targets

## Abstract

Despite tremendous progress made during the last few decades in the treatment options for cancer, compounds isolated from Mother Nature remain the mainstay for therapy of various malignancies. Fangchinoline, initially isolated from the dried root of *Stephaniae tetrandrine*, has been found to exhibit diverse pharmacological effects including significant anticancer activities both in tumor cell lines and selected preclinical models. This alkaloid appears to act by modulating the activation of various important oncogenic molecules involved in tumorigenesis leading to a significant decrease in aberrant proliferation, survival and metastasis of tumor cells. This mini-review briefly describes the potential effects of fangchinoline on important hallmarks of cancer and highlights the molecular targets modulated by this alkaloid in various tumor cell lines and preclinical models.

## 1. Introduction

On a global scale, cancer is considered as the second major disease that causes significant morbidity, making it a major public health concern. Indeed, despite a positive response to initial treatment options, a number of patients may develop local recurrence and propagation of the primary tumor [1]. Cancer may arise as a consequence of genetic dysfunction, amplification, mutations, repression, and deletion of genes [2]. It involves multiple steps such as initiation: Mutations by alteration of the DNA under the influence of carcinogens; promotion: Clonal expansion with formation of a tumor; proliferation: With two often simultaneous steps of spreading of malignant cells in the nearby organs and metastasis and dissemination of cancer cells [3]. In addition, an increase in proliferation combined with a decrease in apoptosis favors an increase in number of actively dividing tumor cells, thereby leading to in situ cancer [4]. Therefore, the design and development of novel agents derived from natural sources that are known to affect multiple molecular and cellular targets is being explored as an effective approach for cancer treatment [5,6].

Natural products and bioactives refer to compounds, exclusive of essential nutrients, that have a biological activity in humans [7,8,9]. They have been historically a mainstay source of anti-cancer drugs. It has been demonstrated that drugs derived from natural compounds may be more effective for cancer treatment than those produced synthetically [10]. Currently, it is known that approximately 10,000 out of 500,000 plant species potentially contain medicinal substances [11]. According to the United States Food and Drug Administration (US FDA), natural products and their derivatives represent one-third of all novel drugs. For cancer therapy, almost 80% of all drugs approved by the US FDA during the last three decades are natural products, their derivatives, or drugs designed based upon a natural product structure. New technologies and analysis methods, such as combinatorial synthesis and high-throughput screening, have brought a fresh perspective for the discovery and the design of novel compounds from natural sources, including medicinal plants [1,12,13,14,15].

A summary of the drugs derived from natural products and then used as active ingredients for anti-tumor drugs approved by the FDA is now provided. Irinotecan, a chemotherapy drug used to treat colorectal cancer, extracted from *Camptotheca acuminata* was approved by the FDA in 1996 [16]. Etoposide, an epipodophyllotoxin, is a chemotherapeutic drug used for the treatment of various human cancers including small cell lung and testicular cancers. It is extracted from *Podophyllum peltatum* and was approved by the FDA in 1983 [17,18]. Masoprocol is used to treat actinic keratoses and was originally extracted from *Larrea tridentata* and was approved by the FDA in 1992 [19]. Teniposide, which is extracted from podophyllum, is used to cure acute lymphocytic leukemia through the inhibition of DNA synthesis by the formation of a complex with topoisomerase II and DNA, which leads to DNA breaks. The accumulation of the DNA fragments can prevent cells from entering the mitotic phase during the cell cycle, and lead to cell death. Teniposide acts primarily in the G2 and S phases of the cell cycle, and its use was approved by the FDA in 1992 [20]. Paclitaxel, which is derived from *Taxus brevifolia*, can induce the polymerization of microtubules thus causing apoptosis and was approved for clinical use by the FDA in 1992 [21]. Docetaxel, a semi-synthetic taxane can also bind to tubulin, thus stabilizing microtubules and inducing apoptosis. Its use was approved by the FDA in 1996 [22]. Finally, vinorelbine, a vinca alkaloid derived from *Catharanthus roseus*, was approved as a medicine for non-small cell lung cancer by the FDA in 1994 [23].

## 2. Fangchinoline Classification

**Figure 1:**
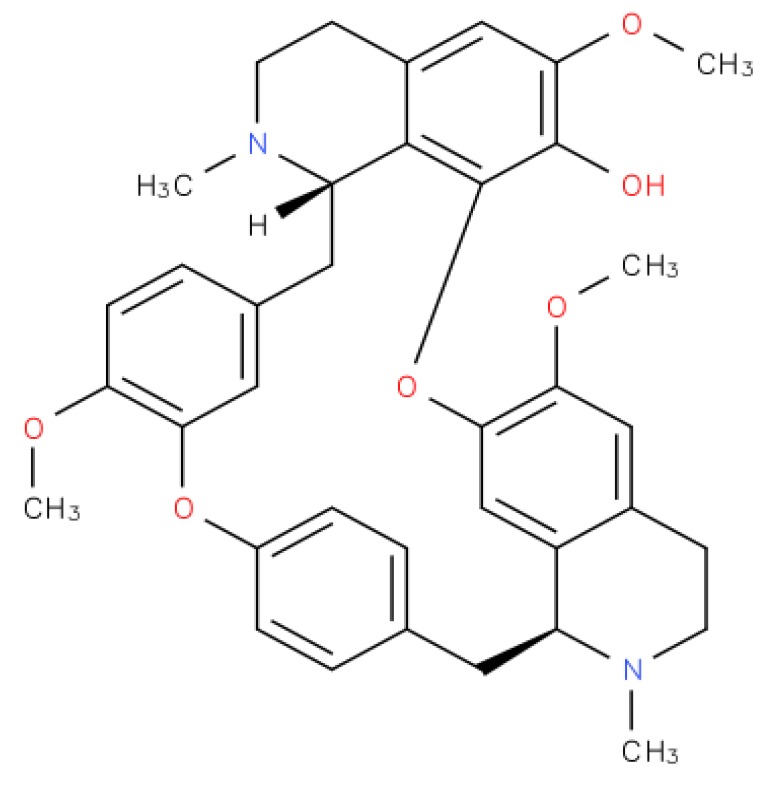
The chemical structure of fangchinoline.

Fangchinoline is a traditional chinese medicine isolated from the dried root of *Stephaniae tetrandrine* S. Moore (family-menispermaceae) [24]. This bisbenzylisoquinoline alkaloid (Figure 1) showed significant anti-cancer effects in several tumor cell lines, including MDA-MB-231 breast cancer cells [25,26,27], MG63 and U20S bone cancer cell lines [28], A549 lung cancer cell lines [29], PC3 human prostate cancer cell lines [30], K562 myelogenous leukemia cells [31], T24 and 5637 bladder cancer cell lines [32], human gastric cancer cells AGS [33], and U87 MG and U118 MG glioblastoma multiforme cancer cell lines [34].

## 3. Fangchinoline-Reported Anti-Cancer Effects in Vitro and in Vivo

### 3.1. Effect on Tumor Cell Proliferation

Proliferation is an important part of tumor development and progression. To multiply, cancer cells short-circuit a number of the regulatory pathways involved in proliferation, allowing them to grow in an uncontrolled manner. These cells have several approaches to avoid cellular senescence [35], which is a phenomenon that allows the limiting of the replicative capacity of cells, thus preventing their proliferation at different stages of malignancy. Fangchinoline has been reported to exhibit potent anti-proliferation effects against several types of tumor cells. Its anti-proliferative activity and effect on various regulators of cell growth has been substantiated in a variety of malignant cells, including bone cancer/osteosarcoma (MG63 and U20S) [28], breast cancer (MDA-MB-231) [25,27], and lung adenocarcinoma (SPC-A-1) [36] by various methods such as 3-(4,5-dimethylthiazol-2-yl)-2,5-diphenyltetra-zolium bromide (MTT) assay, flow cytometric analysis, Western blot, and reverse transcription polymerase chain reaction (RT-PCR) techniques.

Osteosarcoma (also called osteogenic sarcoma) is the most common bone cancer, and it affects mostly children and young adults [37]. Preoperative chemotherapy is the current treatment option, but it has a limited long-term effect to prevent the progression of disease. Fangchinoline was found to significantly decrease the proliferation of MG63 and U20S bone cancer cell lines, along with the suppression of migration of MG63 cells [28]. In MDA-MB-231 as well as SPC-A-1 cells, a time-dependent significant inhibition of cell proliferation has been shown following treatment with fangchinoline [25,36]. A study by Guo et al., on A549 lung adenocarcinoma cell line treated with fangchinoline, revealed the potential of the drug to cause suppression of both proliferation and invasion [29]. In T24 and 5637 bladder cancer cell lines treated with fangchinoline, a concentration-dependent reduction of intracellular ATP levels were associated with a down-regulation of cell proliferation [32]. Additionally, it was found that treatment of the PC3 human prostate cancer cell line with fangchinoline resulted in the attenuation of cell proliferation [30]. Furthermore, fangchinoline can induce a substantial inhibition of cell proliferation in K562 myelogenous leukemia cells derived from the blast crisis of chronic myeloid leukemia [31].

### 3.2. Anti-Metastatic Effects

Metastasis is the leading reason for the resultant mortality of patients with cancer. It represents the end-product of the invasion and metastasis cascade, and involves the dissemination of tumor cells to distant organs followed by their adaptation to the new tissue microenvironments [38]. Melanoma is a tumor with a high degree of malignancy, metastasis, and mortality. The etiology of melanoma has not been fully elucidated, and there is no effective drug for its complete treatment [39]. In a recent study conducted on A375 and A875 melanoma cell lines, it has been shown that fangchinoline could significantly inhibit cell metastasis and migration (IC50 values of 12.41 and 16.20 µM) in a concentration-dependent manner [40] as determined by scratch wound healing and transwell assays.

A glioma is a tumor that starts in the glial cells of the brain or the spine [41]. It can be classified clinically following the grade of the tumor in four grades, from grade I to grade IV, depending on the growth rate [42,43,44,45]. Among various glial tumors, glioblastoma multiforme (GBM, a grade IV glioma) is the most aggressive but also sadly the most common and destructive cancer that occurs in human brains. Indeed, most patients with GBMs have a life expectancy of less than one year, with a short-term survival. Over the last fifty years, GBMs have evaded increasingly clever and intricate attempts at therapy [46]. It has been shown that fangchinoline inhibits tumor cell invasiveness and proliferation in U87 MG and U118 MG GBM cell lines [34]. In AGS human gastric cancer cells, fangchinoline, in a dose-dependent manner, suppressed the adhesion, migration, and invasion of the cells without any obvious cytotoxic effects [33].

### 3.3. Effects on Apoptosis and Autophagy

Apoptosis, also known as the process of programmed cell death, is highly selective; and thus in both physiological and pathological conditions, apoptosis is generally characterized by specific morphological characteristics that concern both the nucleus and the cytoplasm, in which three principal changes at the biochemical level can be observed: Expression of phosphatidylserine (PS) in the outer layer membrane, followed by its recognition by phagocytes, and finally activation of caspases. Defects in apoptosis can cause cancer or autoimmune diseases, while enhanced apoptosis may also lead to various degenerative diseases [4,47,48,49]. 

Autophagy is an intracellular catabolic degradative process, which allows capturing and degradation of intracellular components (proteins, organelles) to sustain homeostasis and metabolism [50]. Over time, one can observe cellular toxicity if there is an accumulation of damaged proteins and organelles in the cells, which is why low levels of basal autophagy play an important role in protein and organelle turnover [51,52]. In cancer, autophagy may have two opposite roles: It can function as a tumor suppressor or as a tumor promoter. This can be explained by the fact that autophagy can be either cytoprotective or cytotoxic through mechanisms that are still not completely clarified [53,54].

Fangchinoline has been shown to induce apoptosis in diverse malignant cells by various techniques (such as Hoechst 33258 staining, Annexin V-fluorescein isothiocyanate or Annexin V-propidium iodide methods) including human U118 and U87 MG grade IV GBM cell lines [34]. Additionally, increased exposure time to fangchinoline caused an up-regulation of apoptosis in PC3 prostate cells [30]. T24 and 5637 bladder cancer cell lines treated with fangchinoline demonstrated increased apoptosis and adaptive autophagy in a concentration-dependent manner [32]. It was found that, in human pancreatic cancer cells, fangchinoline induced apoptosis [55]. Fangchinoline significantly decreased MDA-MB-231 breast tumor cell growth and induced apoptosis in a concentration-dependent manner [56]. In the human liver cancer cell lines PLC/PRF/5 and HepG2, fangchinoline induced autophagy in an irreversible way [57]. Fangchinoline was also found to significantly increase apoptosis in MG63 and U20S bone cancer cell lines [28].

### 3.4. Molecular Targets

Fangchinoline has been observed to exert growth modulatory, pro-apoptotic/autophagic and anti-metastatic effects, as described in the above sections, by modulating a wide variety of different cellular signal transduction cascades. These effects of fangchinoline on a few important molecular targets are briefly discussed in this section. Previous studies have demonstrated that the regulation of cellular growth predominantly depends on a number of tumor suppressors, protooncogenes/oncogenes, and also signaling molecules, such as phosphatidylinositol-3-kinase (PI3K) and protein kinase B (Akt) [58]. PI3K is a lipid kinase that can be activated by receptor tyrosine kinases, leading to an important secondary messenger phosphatidylinositol-3,4,5-trisphosphate production, thus allowing protein kinase B (PKB) to be activated by its translocation to the plasma membrane. PKB (also known as Akt), is a serine/threonine protein kinase [59]. Its phosphorylation by phosphoinositide-dependent kinase (PDK) 1 and 2 of two of its residues (threonine 308 (Thr308) in the activation loop and serine 473 (Ser473) in the C-terminal hydrophobic motif) induces its activation [60,61]. PKB plays a key regulatory role in cell survival and proliferation [62]. This role includes the inactivation of pro-survival factors, including glycogen synthase kinase-3-beta (GSK-3β) [63]. In addition, it has been demonstrated that PKB phosphorylates the cell cycle inhibitory protein p21(Cip1) at Thr 145, which inhibits DNA replication [64], as well as Bcl-2-associated death promoter (BAD), a member of the BCL-2 family, thus permitting the promotion of cellular survival [65]. It has been shown that PI3K-Akt activation is required for the induction of membrane type 1 metalloproteinase (MT1-MMP) [66,67], a transmembrane MMP that plays a major role in extracellular matrix remodeling; directly by degrading several of its components and indirectly by activating pro-MMP2, an ubiquitinous MMP that is involved in diverse functions such as angiogenesis and tumor invasion, as well as degradation of extracellular matrix proteins [68]. MT1-MMP can also up-regulate the expression of vascular endothelial growth factor expression and thus promote tumor growth and angiogenesis [69].

Extracellular matrix interactions with cells play a critical role in tumor metastasis [70]. Focal adhesion kinase (FAK) is an ubiquitous cytoplasmic tyrosine kinase known as a key mediator of signaling by integrins, which are the principal receptors on animal cells for binding most extracellular matrix proteins including laminins, growth factors, collagens, and fibronectin [71,72]. MMP-9, which is involved in the degradation of the extracellular matrix, plays an important role in tumor invasion and metastasis, and its expression is regulated by various growth factors, including TGF-β1, in different cell types [73]. In integrin-mediated cell adhesion, FAK is activated through the disruption of an auto-inhibitory intra-molecular interaction between its amino terminal FERM (F for 4.1 protein, E for ezrin, R for radixin and M for moesin) domain and the central kinase domain. The activated FAK forms a complex with Src family kinases, initiating multiple downstream signaling pathways through phosphorylation of other proteins to regulate different cellular functions including metastasis [71]. It has been shown that FAK is over-expressed in several cancer cell lines, including human colorectal cancer [74,75]. A number of alterations in various signal transduction cascades associated with metastasis including FAK have been observed following treatment of several cancer cell lines with fangchinoline as highlighted below.

The MAPK/ERK pathway (also called the Ras-Raf-MEK-ERK pathway) is a FAK downstream pathway that includes the expression of G protein (RAS) and protein kinases (RAF, MEK, ERK1 and 2). After the binding of a ligand to the membrane receptor tyrosine kinase, a signal is communicated, causing the translocation of ERK (MAPK) to the nucleus, where it activates transcription factors that result in gene expression [76]. In MDA-MB-231 breast cancer cells treated with fangchinoline, it has been demonstrated that inhibition of cell proliferation was induced by the activation of apoptosis via the mitochondrial apoptotic pathway, and that decreased levels of phosphorylated Akt were associated with a decreased effect of fangchinoline [25]. Fangchinoline was also found to decrease PI3-K activation and expression of its downstream signaling pathways, cyclin D1, and matrix metalloproteinase 2 and 9, in addition to decreasing Akt (phospho-Thr308) expression. It also caused an up-regulation of the expression of caspase-3 and caspase-8 (a family of endoproteases that initiates the process of apoptosis) in MG63 and U20S cells [28].

In U87 MG and U118 MG cells, it has been shown that fangchinoline decreases the kinase activity of Akt and deletes its phosphorylation at Thr308 and Ser473 through suppressing the Akt-mediated signaling cascades, including Akt/p21, Akt/Bad, and Akt/MMPs [34]. Fangchinoline, in a dose-dependent manner repressed the phosphorylation of Akt and the expression of MMP-2 and 9 at both the mRNA and protein levels, and caused an increased expression of tissue inhibitor of metalloproteinase TIMP-1 and -2 mRNAs in AGS cells [33]. In MDA-MB 231 cells, long exposure times to fangchinoline induced a decrease of the levels of MMP-2 and -9 as well as the expression of NF-κB protein, while the expression of NF-κB transcription factor inhibitor (IκB) was found to be increased [56]. 

In the A549 lung adenocarcinoma cell line, fangchinoline inhibited the phosphorylation of FAK (Tyr397) and its downstream pathways, the FAK-Akt pathway and FAK-MEK-ERK1/2 [29]. In another recent study, it was noted that fangchinoline exerted its anticancer effects on human melanoma (A375 and A875) cells by attenuating the phosphorylation of FAK. It was observed that this alkaloid suppressed the proliferation of cells and metastasis in vitro via decreasing cyclin D1 and paxillin expression respectively. Moreover, pre-treatment with pharmacological FAK inhibitor (PF-562271) significantly abolished the growth inhibitory as well as anti-metastatic effects, thus establishing FAK as one of the key kinases mediating the observed anti-neoplastic effects of fangchinoline in the melanoma cells [40]. In hepatocellular carcinoma cell lines, HepG2 and PLC/PRF/5, fangchinoline was found to dose-dependently induce autophagy instead of apoptotic cell death. Nuclear translocation of p53 was involved in induction of autophagy by fangchinoline, followed by selective transactivation of the autophagy-related gene sestrin 2 and initiation of the autophagic process. Signaling by the AMP-activated protein kinase was also involved as a downstream target of sestrin 2 and induced rapamycin (mTOR) independent autophagic cell death in both cell lines. It has been previously demonstrated that, both at a genetic or protein level, a dysregulation of mTOR and its downstream targets can contribute to malignant tumorigenesis [57,77]. 

In T24 and 5637 cells treated with fangchinoline, it has been shown that the level of cleavage of caspase-3 is increased, in addition to an up-regulation of the LC3-II/LC3-I ratio and down-regulation of p62 level in a concentration-dependent manner, in addition to the inhibition of mTOR and reduction of the intracellular ATP levels [32]. Additionally, it was found that treatment of PC3 prostate cancer cell lines with fangchinoline leads to an increase in protein p27 expression, the inhibition of cyclin-regulated signaling pathways associated with the arrest of the G1/S phase, and inhibition of cyclin D and proliferating cell nuclear antigen expression [30]. In K562 cells derived from the blast crisis of chronic myeloid leukemia, fangchinoline induced cell cycle arrest at the G0/G1 phase through the up-regulation of cyclin-dependent kinase (CDK)-N1A and MCL-1 mRNA levels, as well as the down-regulation of cyclin D2 mRNA levels [31]. Fangchinoline, in a dose-dependent manner, arrested the cell cycle at the G0/G1 phase in SPC-A-1 lung adenocarcinoma cells, which was associated with a decrease in their proliferation [36]. It has been shown in several studies that the orphan nuclear receptor subfamily 4 group A member 1 of the nerve growth factor IB (NR4A1) is over-expressed in human pancreatic cancer, and antagonizing this receptor promotes apoptosis and inhibits pancreatic tumor cell growth [55]. In human pancreatic cancer cells, fangchinoline inhibited cell proliferation and induced apoptosis, in part through the NR4A1-dependent pro-apoptotic pathways. It also decreased the expression of the anti-apoptotic protein, survivin, by inhibiting 1 (Sp1)-mediated transcription and caused oxidative stress-mediated endoplasmic reticulum (ER) stress in pancreatic cancer cells [55]. In human breast cancer cell lines, MCF-7 and MDA-MB-231, it has been shown that fangchinoline significantly inhibited the expression of cyclins D1, 3, and E, the kinase activities of CDK2, 4, and 6 (which are over-expressed in breast cancer) and increased the expression of cyclin-dependent kinase (CDK) inhibitors p21/WAF1, and p27/KIP1 [27,78]. 

### 3.5. Effects on Multi-Drug Resistance and Other Parameters

Multi-drug resistance (MDR) has become the largest obstacle to the success of cancer chemotherapies and other autoimmune diseases. It is mediated by various cellular pathways, some of which remain to be elucidated [79]. The results of a recent study conducted in vivo in rats, as well as in vitro, suggest that fangchinoline has a protective effect against the liver toxicity induced by paracetamol/carbon tetrachloride, and it may be due to the synergistic action of fangchinoline through free radical scavenging that prevents oxidative stress [80]. 

Chronic myeloid leukemia (CML) is a hematopoietic stem cell disease caused by the oncoprotein BCR-ABL, which has a main tyrosine kinase activity. So far, the first-line therapy for CML is imatinib mesylate (IM), which has an inhibitory effect on the tyrosine kinase activity of BCR-ABL. Unfortunately, in a number of patients, IM has a lower efficiency in the advanced stages of CML due to the apparition of mutations that amplify the BCR-ABL gene expression and thus the development of IM resistance. Fangchinoline was found to induce cell cycle and proliferation arrest of the blast crisis, thus by passing the resistance mechanism, which suggests that fangchinoline may be a potential effective anti-tumor agent against CML [31].

A particular class of transmembrane glycoproteins (P-glycoprotein; P-gp) functions as an energy-dependent drug-efflux pump, and thus induces multi-drug resistance to cancer treatment [81,82]. A recent study was conducted in vitro in MDR1-MDCK II cells, which originate from transfection of Madin Darby canine kidney MDCK cells with the *MDR1* gene that encodes for the efflux protein, P-gp. It was shown that fangchinoline decreased the efflux of paclitaxel and inhibited the multi-drug resistance of the drug mediated by P-gp [83,84]. It has also been reported previously that fangchinoline, along with another alkaloid tetrandrine, can exert a synergistic cytotoxic effect in MDR Caco-2 and CEM/ADR5000 cancer cells in conjunction with doxorubicin treatment. Interestingly, both tetrandrine and fangchinoline augmented the intracellular accumulation of the fluorescent P-gp substrate rhodamine 123, inhibited its efflux and significantly reduced P-gp expression in a concentration-dependent manner [85]. The results of another study provided evidence that fangchinoline can act as a Ca^2+^ channel blocker, and then mitigate the harmful effects of cyanide-induced neuronal cell death by inhibiting glutamate release and oxidants generation. It also interfered with the increase in Ca^2+^ concentration observed due to the harmful effects of H_2_O_2_-induced neuronal cell death, and inhibited glutamate release as well as the generation of reactive oxygen species [86]. Overall, the various molecular targets modulated by fangchinoline are briefly summarized in Table 1.

### 3.6. In Vivo Effects

In PC-3 tumor-bearing nude mice treated with fangchinoline, an induction of apoptosis was observed in addition to an inhibition of tumor growth and a decrease in proteasome activities in tumor xenografts in both a dose- and time-dependent manner [88]. 

Fangchinoline was also found to exert an antidepressant-like action in mice via acting at multiple molecular targets [89]. Interestingly, Deng and coworkers reported that fangchinoline significantly abrogated the tumor growth in breast cancer xenograft mouse model by modulating the expression of various oncogenic biomarkers including CD117 and Ki-67 [87]. In addition, it has been found that fangchinoline can effectively attenuate the growth of subcutaneous osteosarcoma tumors in Balb/c mice through the modulation of activation of PI3K and its associated signal transduction pathways [28]. However, additional preclinical studies are required to validate the potential anti-antiangiogenic and anti-metastatic effects of fangchinoline in suitable mouse models.

### 3.7. Effects of the Derivatives of Fangchinoline

In P388/ADR mouse lymphoma cancer cell lines, it has been shown that an addition of a derivative of fangchinoline to vinblastine significantly enhanced its anticancer potency. This effect was greater in comparison with verapamil, another cancer drug that acts through the inhibition of calcium channels and was found to be mediated by increase in the intracellular levels of vinblastine, thereby leading to its accumulation in P388/ADR cells as well as prolonging the life-span of P388/ADR-bearing mice [81,82].

## 4. Conclusions 

**Figure 2:**
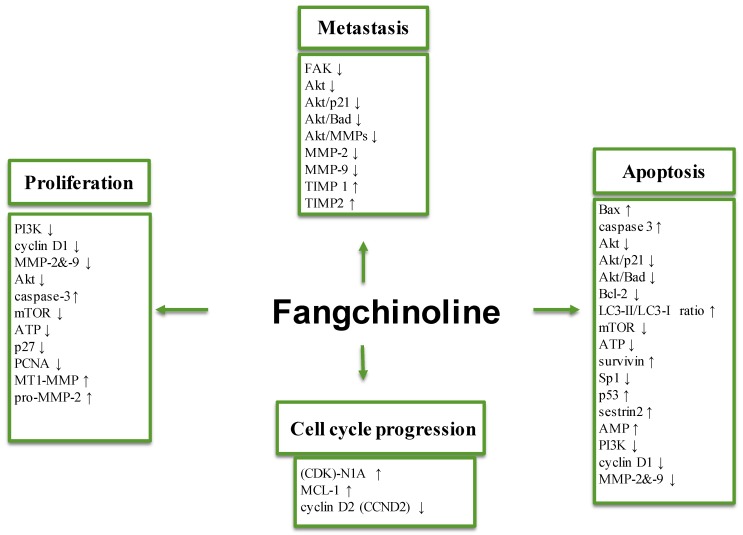
Molecular targets modulated by fangchinoline in tumor cells. FAK: Focal adhesion kinase; MMP2/MMP9: Matrix metalloproteinase; Akt: Protein kinase B; MEK: Mitogen-activated protein kinases; ERK: Extracellular signal-regulated kinases; PI3K: Phosphoinositide 3-kinase; NF-κB: nuclear factor kappa-light-chain-enhancer of activated B cells; Iκβ protein: NF-κB transcription factor inhibitor; LC3: Microtubule-associated protein 1A/1B-light chain 3; p62: Nucleoporin p62; mTOR: Mechanistic target of rapamycin; p27: Cyclin-dependent kinase inhibitor; BAD: Bcl-2-associated death promoter; NR4A1: Nuclear receptor subfamily 4 group A member 1 of the nerve growth factor IB; Sp1: Specificity protein 1; AMP: Adenosine monophosphate; TIMP: Metallopeptidase inhibitor 1. ↑: Upregulation. ↓: Downregulation.

This mini-review briefly summarizes the pleiotropic anti-cancer properties of fangchinoline. In vitro and in vivo findings have identified few important molecular and cellular targets, as well as the mechanisms of action by which the anti-cancer effects (inhibition of tumor cell proliferation, metastasis, and induction of cell apoptosis and autophagy) of fangchinoline and its few derivatives could be exerted. Fangchinoline was also observed to have an inhibitory effect on important oncogenic pathways (FAK, MEK-ERK1/2, NF-κB, PI3K-Akt-mTOR) in diverse tumor cells (Figure 2). Finally, the significant anti-tumor potential of fangchinoline against tumor cell lines together with the significant efficacy observed in preclinical models of selected cancers, makes a case for further preclinical as well as clinical studies of its use as a novel therapeutic agent for the treatment of malignancies. 

## Figures and Tables

**Table 1:** Molecular targets modulated by fangchinoline in in vitro and in vivo models of cancer.

Cancer Types	Concentration Range Tested	Pathways/Molecules Altered	References
A549 lung adenocarcinoma	10–40 µM	FAK ↓; FAK-paxillin/MMP-2&-9 ↓; FAK-Akt ↓; FAK-MEK-ERK1&2 ↓	[29]
MG63 and U20S bone cancer	10–30 µM	PI3K ↓; Cyclin D1 ↓; MMP2&9 ↓; Akt ↓; Caspase3 &8 ↑	[28]
MDA-MB-231 breast cancer	6.25–100 µM	MMP-2&-9 ↓ Expression of NF-κB ↓ Phosphorylated Akt ↓ IκB protein ↑	[25,56,87]
SPC-A-1 lung adenocarcinoma	2.5–10 µM	G0/G1 phase Arrest ↓	[36]
T24 and 5637 bladder cancer	2.5–40 µM	Caspase-3 ↓ LC3-II/LC3-I ratio ↑ p62 ↓ mTOR ↓ Intracellular ATP level ↓	[32]
PC3 prostate cancer	10–30 µM	G1/S phase ↓ Cyclin-D ↓ PCNA ↓ p27 expression ↑	[30]
U87MG and U118MG GBM	10–30 µM	Akt/p21 ↓ Akt/BAD ↓ Akt/MMP2&9↓	[34]
HepG2 and PLC/PRF/5 hepatocellular carcinoma	2–10 μM	p53 ↑ Sestrin2 ↑ AMP ↑	[57]
AGS gastric cancer	0–60 μM	Akt↓ MMP2&9 ↓ TIMP1&2 ↑	[33]
A375 and A875 melanoma	10–20 μM	FAK ↓	[40]
MiaPaCa-2 and Panc-1 pancreatic cancer	0–15 μM	NR4A1 ↓ Survivin ↓ Sp-1 ↓	[55]

FAK: Focal adhesion kinase. MMP2&9: Matrix metalloproteinases. Akt: Protein kinase B. MEK: Mitogen-activated protein kinases. ERK: Extracellular signal-regulated kinases. PI3K: Phosphoinositide 3-kinase. NF-κB: Nuclear factor kappa-light-chain-enhancer of activated B cells. IκB protein: NF-κB transcription factor inhibitor. LC3: Microtubule-associated protein 1A/1B-light chain 3. p62: Nucleoporin p62. mTOR: Mechanistic target of rapamycin. ATP: Adenosine triphosphate. PCNA: Proliferating cell nuclear antigen. p27: Cyclin-dependent kinase inhibitor 1B. BAD: Bcl-2-associated death promoter. NR4A1: Nuclear receptor subfamily 4 group A member 1 of the nerve growth factor IB. Sp-1: Specificity protein 1, p53: Phosphoprotein, cellular tumor antigen. AMP: Adenosine monophosphate. TIMP: Metallopeptidase inhibitor 1. ↑: Upregulation. ↓: Downregulation.
